# 多孔框架材料用于食品中新污染物的样品前处理研究进展

**DOI:** 10.3724/SP.J.1123.2026.01023

**Published:** 2026-10-08

**Authors:** Jiaqi HUANG, Yangxia HAN, Wei HA, Yingshan ZHANG, Yanping SHI

**Affiliations:** 1. 中国科学院兰州化学物理研究所，甘肃 兰州 730000; 1. Lanzhou Institute of Chemical Physics，Chinese Academy of Sciences，Lanzhou 730000，China; 2. 西藏奇正藏药股份有限公司，甘肃 兰州 730011; 2. Tibet Cheezheng Tibetan Medicine Co. ，Ltd. ，Lanzhou 730011，China; 3. 中国科学院大学，北京 100049; 3. University of Chinese Academy of Sciences，Beijing 100049，China

**Keywords:** 多孔框架材料, 新污染物, 样品前处理, 食品分析, porous framework materials, emerging pollutants, sample pretreatment, food analysis

## Abstract

近年来，新污染物因具有环境持久性、生物累积性和毒性而备受关注。膳食摄入是新污染物进入人体的主要途径之一，因此，为准确评估其膳食暴露风险，复杂食品基质中痕量新污染物的检测技术面临更高要求。传统的分析技术已难以满足当前快速、精准的分析需求，因此亟需发展高效的样品前处理技术。多孔框架材料以其可调孔隙、高比表面积、易功能化修饰等优势，为新污染物的样品前处理提供了理想的材料平台。本文综述了金属有机框架（MOFs）、共价有机框架（COFs）和氢键有机框架（HOFs）3类多孔框架材料，重点阐述了其通过理性设计提升吸附性能的策略及其在食品中新污染物萃取富集中的应用进展，并对该领域未来的发展方向进行了展望，以期为开发高效、实用的样品前处理新材料与方法提供参考。

## Abstract (en)

Emerging pollutants （ECs） generally refer to chemicals or microorganisms that have known potential toxicity and health hazards. The defining characteristics of ECs include environmental persistence， bioaccumulation and biological toxicity. Primary categories of ECs comprise endocrine disrupting chemicals （EDCs）， persistent organic pollutants （POPs） and antibiotics. Dietary intake represents a predominant pathway for human exposure to ECs. This necessitates the development of highly sensitive and reliable analytical methods. Conventional analytical instruments are fundamental for ECs determination. These include high performance liquid chromatography （HPLC）， liquid chromatography-tandem mass spectrometry （LC-MS/MS）， and gas chromatography-tandem mass spectrometry （GC-MS/MS）. However， the direct analysis of ECs in food remains a significant challenge. This is due to their wide sources， numerous types， complex structures， and ultra-low concentrations alongside substantial matrix interferences. Consequently， it is urgent to develop efficient sample pretreatment techniques to extract， enrich and purify ECs in food， in order to reduce matrix interference and construct the foundation for establishing highly sensitive， accurate， and stable analytical methods. Among various pretreatment techniques， solid-phase extraction （SPE） has gained prominence. It offers notable advantages over liquid-liquid extraction （LLE）. These advantages include simple operation， low consumption of organic solvents and higher enrichment factors. To address specific analytical needs， several advanced SPE modalities have been developed， including magnetic solid-phase extraction （MSPE）， solid-phase microextraction （SPME）， dispersive solid-phase extraction （dSPE）， and stir bar sorptive extraction （SBSE）. Each variant offers unique benefits in terms of automation， speed， or selectivity. The performance of any SPE-based method is fundamentally governed by the adsorbent material. In recent years， porous framework materials have emerged as a revolutionary class of sorbents. They offer exceptional properties for extraction technology. Porous framework materials mainly include metal organic frameworks （MOFs）， covalent organic frameworks （COFs）， and， hydrogen-bonded organic frameworks （HOFs）. These materials are distinguished by their high specific surface area， tunable pore size and abundant sites for functionalization. Their structural periodicity and designability enable precise interaction with target analytes. This review provides a comprehensive and systematic overview of recent progress. It focuses on MOFs， COFs， and HOFs as next-generation adsorbents for ECs analysis. The article begins by introducing the fundamental structures and general properties of these three material classes. It details the synthesis strategies for both pristine materials and their composites. A major emphasis is placed on rational design principles. These principles aim to enhance adsorption performance. Key strategies discussed include ligand functionalization， defect engineering， and the construction of composite/hybrid materials. The review explains how these strategies modulate the adsorption mechanism， including size-exclusion， electrostatic interaction， *π*-*π* stacking， hydrogen bonding， and coordinative interaction. Subsequently， the application progress of these designed materials is thoroughly summarized. It is organized according to major ECs categories： EDCs， POPs， and antibiotics. For each category， representative studies from the past three years are highlighted. The discussion covers material design， extraction format （MSPE， dSPE， SBSE， SPME， etc.）， analytical technique coupling， and key performance metrics. Metrics include adsorption capacity， selectivity， and reusability. The successful determination of ECs in complex food samples is demonstrated. Finally， the review critically examines significant outstanding challenges and suggests corresponding directions for future research. These include the long-term stability of some frameworks in complex matrices. Scalable and green synthesis methods are also needed. Furthermore， seamless integration of these materials into automated， online analytical platforms requires development. Future research should prioritize several areas. Deeper mechanistic understanding via in-situ characterization and computational modeling is essential. The development of robust， environmentally friendly synthesis pathways is crucial. Enhancing material stability and selectivity for real-world applications is a key goal. Finally， translating powdered materials into practical， engineered devices is an important step. In conclusion， porous framework materials hold immense promise. They are poised to redefine sample pretreatment for food safety analysis. This review aims to serve as a valuable reference. It is intended for researchers developing new adsorbent materials and analytical methods for ECs.

新污染物泛指具有已知或潜在毒性和健康危害的化学品与微生物^［1］^，目前备受关注的新污染物包括内分泌干扰物（endocrine disrupting chemicals， EDCs）、持久性有机污染物（persistent organic pollutants， POPs）及抗生素等^［2］^。新污染物具有环境持久性、生物累积性和毒性，可经由工业生产、畜牧养殖及日常生活等多种途径进入环境，并通过生物累积和食物链传递对人类健康产生危害^［3，4］^。为实现食品中新污染物的精准识别与风险评估，发展高效、高灵敏的检测技术具有重要意义。这不仅是识别潜在健康危害、科学制定食品安全限量标准的基础，更是构建预警体系、实施源头管控、最终阻断新污染物暴露链条的关键环节，对于保障食品安全和公众健康至关重要^［5，6］^。

常见的新污染物检测技术主要有高效液相色谱法（HPLC）^［7-9］^、高效液相色谱-串联质谱法（HPLC-MS/MS）^［10-12］^、气相色谱-串联质谱法（GC-MS/MS）^［13，14］^等。然而，由于新污染物来源广泛，结构复杂且浓度较低，在复杂的食品基质中直接检测新污染物困难较大。因此，亟需开发高效的样品前处理技术，完成食品中新污染物的提取、富集与净化，以降低基质干扰，从而为建立高灵敏度、高准确度且稳定可靠的分析方法奠定基础^［15，16］^。目前，在新污染物的分析中，样品前处理技术的核心仍以液-液萃取（LLE）和固相萃取（SPE）这两类经典与通用的方法为主^［17-19］^。LLE利用目标分析物在两种互不相溶的溶剂中的液体分配系数差异实现分离和富集，但存在有机溶剂消耗大、操作烦琐的弊端。SPE通过固体吸附剂完成目标分析物的选择性吸附后，通过有机试剂将其洗脱，从而达到分离和富集目标分析物的目的。相较而言，SPE具有操作灵活、富集倍数高、有机溶剂消耗少等优点。目前已经有多种SPE衍生模式应用于新污染物的样品前处理中，包括磁性固相萃取（MSPE）^［20，21］^、固相微萃取（SPME）^［22］^、分散固相萃取（dSPE）^［23，24］^和搅拌棒吸附萃取（SBSE）^［25］^等。吸附剂是SPE的核心，其性能直接决定萃取效率。传统吸附剂因选择性与特异性不足，难以满足复杂食品基质中痕量新污染物的高灵敏、高选择性分析需求。因此，开发兼具稳定物理化学性质、高富集能力和选择性的新型吸附材料，是实现食品中痕量新污染物精准高效检测的关键。

近年来，多孔框架材料在萃取领域受到广泛关注。多孔框架材料包括金属有机框架（metal organic frameworks， MOFs）、共价有机框架（covalent organic frameworks， COFs）和氢键有机框架（hydrogen-bonded organic frameworks， HOFs）等。它们凭借孔隙率高、孔径可调、比表面积大、修饰位点丰富等卓越特性，作为吸附剂表现出优异的吸附性能和高选择性，已被广泛应用于不同场景下的新污染物分析检测中^［26-28］^。基于此，本文系统综述了用于食品中新污染物分析的常见多孔框架材料。首先，依据材料类型进行分类，重点阐述了通过理性结构设计与功能精准调控提升其吸附性能的最新策略。其次，结合几类典型的新污染物，详述了该材料在样品前处理中的最新应用进展。最后，深入探讨了该领域当前面临的挑战与未来的发展机遇。

## 1 多孔框架材料的构筑

多孔框架材料根据构筑单元和连接化学键的差异，主要分为MOFs、COFs和HOFs 3类。本部分归纳了上述3类材料及其复合材料的研究进展，重点总结通过理性结构设计与功能精准调控，以增强其在复杂基质中对新污染物吸附性能的前沿构筑策略。

### 1.1 金属有机框架及其复合材料

MOFs是一类由金属离子/金属簇与有机配体通过配位键自组装连接而形成的晶态多孔材料，于1995年由Yaghi团队报道^［29］^。常见的MOFs系列有ZIFs、UiOs和MILs等。MOFs具有比表面积大、孔隙率高、结构和功能可设计性强的特点。近年来，利用调制剂诱导缺陷形成的策略是实现MOFs结构动态调控与性能优化的前沿方向。该策略通过引入可控缺陷，能精准调制其孔隙结构和活性位点^［30］^。Han等^［31］^通过该策略制备了一系列层级多孔锆基MOF（HP-NU-902-X）。通过改变丙酸的浓度，系统调整HP-NU-902-X的晶体结构从而形成更多的结构缺陷（图1）。这些缺陷不仅使HP-NU-902-X的孔隙率显著增加，还导致在缺失的连接子位点上产生更多的活性吸附中心（-OH/H_2_O基团和Zr链段），从而促进了其与目标分析物磺胺类抗生素的结合。最后，以HP-NU-902-80为吸附剂开发了一种用于富集和去除牛奶和水中的微量磺胺类抗生素的dSPE方法。该策略为制备缺陷型MOFs提供了新思路，并在磺胺类抗生素的样品前处理中得到了应用，证实了其在新污染物的富集和去除方面的应用潜力。此外，研究者通过建立污染物分子结构与MOFs性能间的构效关系，对MOFs的结构进行定向功能设计，以定制针对特定新污染物的高性能吸附剂。例如，Grancha等^［32］^报道了一种基于L-组氨酸衍生的MOF材料Cu^Ⅱ^_2_（*S*，*S*）-hismox·5H_2_O（MOF 1），用于高效捕获水中的短链和长链全氟和多氟烷基物质（PFAS）。该材料具有由疏水配体骨架与亲水Cu（Ⅱ）中心/咪唑基团构成的双亲性纳米通道，同时兼具开放金属位点、良好的生物相容性与稳定性。将MOF 1作为SPE吸附剂，研究了其对多种代表性PFAS的吸附能力。实验结果表明，MOF 1对长链PFAS实现了80%~100%的去除率，对传统材料难以捕获的短链PFAS也实现了70%~86%的去除率，且展现出极快的吸附动力学特征和良好的重复性。通过X射线单晶衍射，直接观测到全氟丁酸（PFBA）和全氟辛烷磺酸盐（PFOS）在MOF中的配位模式，证明了吸附过程是由PFAS的羧基/磺酸基与Cu（Ⅱ）中心配位、卤键及氢键等作用引起的。该研究展现了L-组氨酸衍生的MOF在高效、快速去除PFAS尤其是短链PFAS方面的巨大潜力，为开发高性能的新污染物吸附剂提供了设计思路。

**图1:**
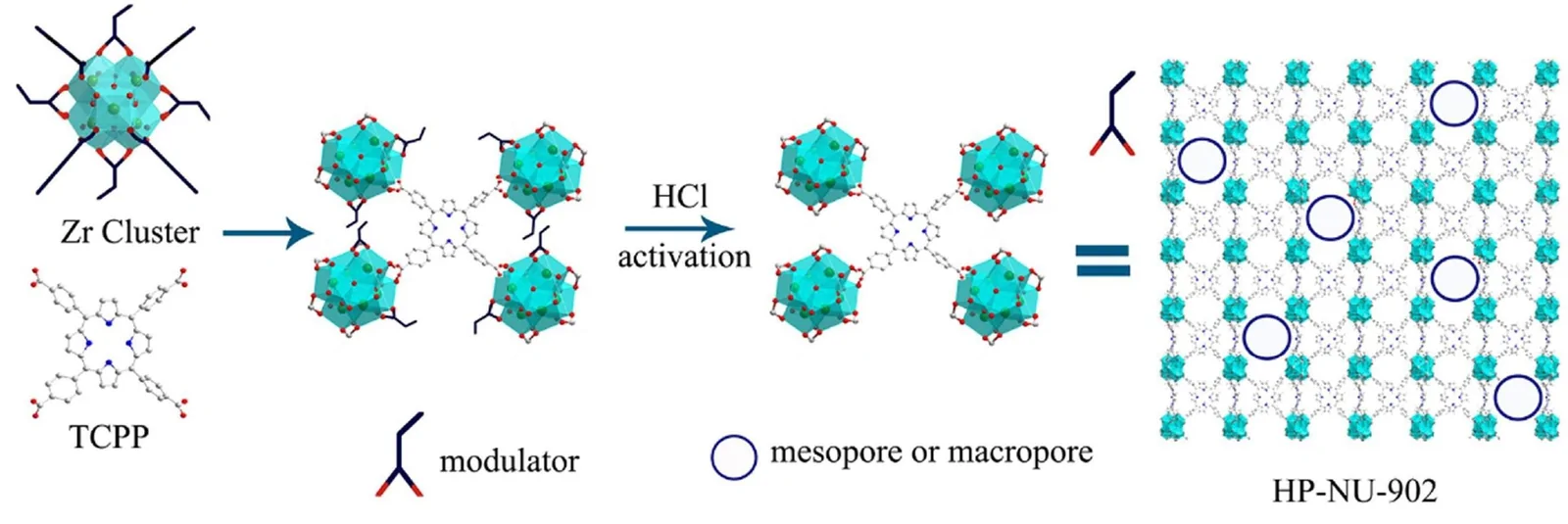
HP-NU-902的制备流程图^［31］^

MOFs复合材料指以MOFs为基体或增强相，与具有不同性能的物质形成的两相或多元相复合材料。它在保留MOFs材料固有结构优势的同时，整合了其他材料的优异性能。其中，具有层次结构的多维MOF-on-MOF复合材料尤为引人关注，它能通过组分间的协同效应，展现出超越单一MOFs的卓越吸附性能等优势^［33］^。Qin等^［34］^通过调控2D-MOF（Zr-BTB）纳米片在3D-MOF纳米片（PCN-134）表面和边缘的原位定向生长，成功制备了仙人掌状2D-3D MOF@MOF材料（PCN-134@Zr-BTB）（图2）。吸附实验表明，PCN-134@Zr-BTB复合材料对4种双酚类化合物（BPs）的吸附容量高达135.1~628 mg/g，性能优于单一组分。通过分析傅里叶变换红外光谱和X射线光电子能谱数据，证明了主要的吸附机制为氢键相互作用和*π*-*π*堆积作用。随后，建立了一种PCN-134@Zr-BTB-dSPE方法用于分析实际水样中的BPs，在湖水中检测到质量浓度低至0.8 μg/L的双酚AF。通过加标回收试验评估了方法的准确性。结果显示，该方法回收率为72.8%~108%，富集因子为310~370，RSD为3.24%~12.7%，表明该方法在实际水样中具有良好的适用性与可靠性。Ilić等^［35］^以优化UiO-66界面为核心，揭示了PFAS去除中功能化界面的重要性，并提出了一种新颖的设计思路。通过系统功能化（X=H、NO_2_、NH_2_、（F）_4_）并结合有机硅（OS）表面涂层，制备了一系列UiO-66-X和UiO-66-X-OS材料。其中，UiO-66-（F）_4_-OS的性能最优，可在ppb级的初始浓度下高效去除全氟辛酸（PFOA）、PFOS等长链PFAS。实验结果表明，通过表面功能化可显著提升材料对不同PFAS的选择性吸附性能，其中UiO-66-（F）_4_及其有机硅涂层复合材料对PFOS等长链PFAS吸附效果尤为突出。此外，通过傅里叶变换红外光谱、高角环形暗场像-扫描透射电子显微镜-X射线能谱仪联用技术表征以及分子动力学模拟，揭示了PFAS分子与MOF表面功能基团的特异性相互作用，而非传统的孔隙捕获作用。研究提出了一种“非孔隙依赖”的吸附机制，即通过优化外部界面性质（如引入疏水涂层、暴露Zr离子位点）来提升PFAS的去除效率。该研究不仅为MOFs吸附剂的设计提供了新思路，也为当前PFAS污染问题提出了解决方案。

**图2:**
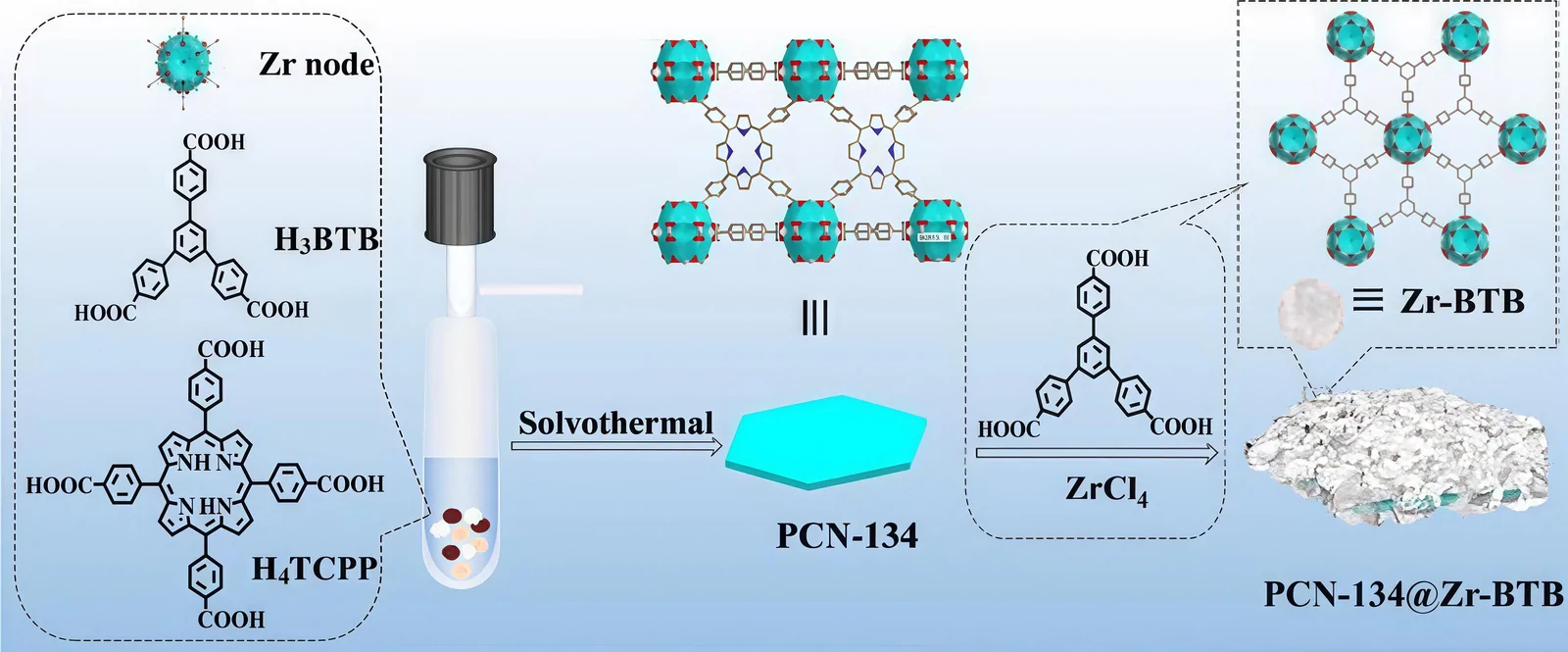
PCN-134@Zr-BTB的制备流程图^［34］^

### 1.2 COFs及其复合材料

COFs是通过可逆共价键连接而成的结晶多孔聚合物，常见的COFs材料有三嗪基COF、亚胺基COF和硼基COF。COFs具有高孔隙率、可调的周期性孔道结构以及明确的功能框架，在吸附领域中具有显著的优势。通过理性设计，在COFs骨架中定向引入特定官能团，使其与目标分子之间形成氢键相互作用、静电相互作用、*π*-*π*堆积作用或卤键相互作用等从而显著提升其对目标分子的吸附能力和选择性。Zhou等^［36］^以4，4′，4″-（1，3，5-三嗪-2，4，6-三基）三（1，1′-联苯）三苯胺（Ttba）和三醛基间苯三酚（Tp）作为配体，制备了一种孔径与BPs相匹配的COFs材料（TpTtba-COF），并将其用作吸附剂，建立了一种用于环境水样中3种BPs的dSPE-HPLC/UV分析检测方法（图3）。该方法具有良好的线性范围（1~800 μg/L）和较低的检出限（0.20~0.32 μg/L），证明通过合理设计COFs以实现高性能BPs吸附与灵敏检测的可行性。

**图3:**
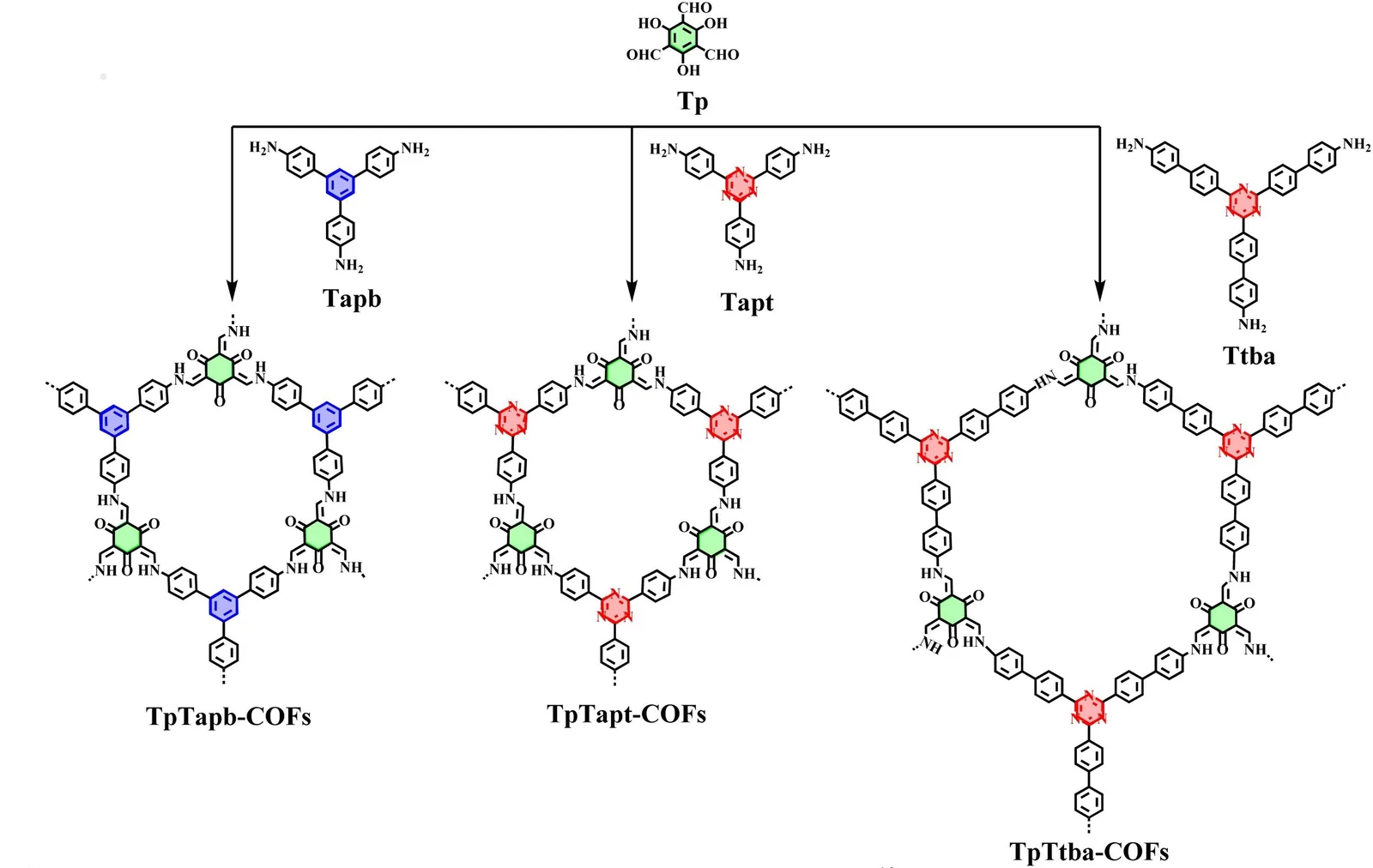
TpTtba-COFs、 TpTapt-COFs和TpTapb-COFs的制备流程图^［36］^

尽管COFs在样品前处理中表现出优异的吸附潜力，但其实际应用仍受限于合成挑战，如结晶度不均一、存在结构缺陷或非晶相，这些问题会显著影响其孔隙率和比表面积，进而影响其吸附性能。因此，将COFs与其他材料通过化学或物理的方法复合已成为关键策略。该策略能在保留COFs固有优势的同时，引入其他材料的优势。目前，磁性COFs、MOF/COFs、MXene/COF等复合材料已广泛应用于MSPE、dSPE、SPME等多种样品前处理技术中，以应对新污染物分析^［37-40］^。例如，Tao等^［41］^通过Schiff-Base反应设计制备了一种具有核壳结构的磁性COF（Fe_3_O_4_@Tp-3F），该材料具有比表面积大、孔隙均匀和磁响应度强的优势。以Fe_3_O_4_@Tp-3F为MSPE吸附剂，开发了一种灵敏分析PFAS的方法（MSPE-HPLC-MS/MS）。该方法具有优良的分析性能：线性范围宽（1~800 ng/L）、检出限低（0.23~1.2 ng/L），并能有效用于实际水样中痕量PFAS的检测。通过密度泛函理论（DFT）计算验证了吸附机制为PFAS与Fe_3_O_4_@Tp-3F之间的氢键相互作用和卤键相互作用，并且随着PFAS链长的增加，卤键作用更加明显。上述结果表明，通过理论计算的方法可以模拟目标污染物与材料之间的特异性相互作用，从而为吸附材料的设计提供理论基础。Gan等^［42］^通过将具有丰富微孔结构的刚性MIL-68（In）与具有介孔结构的COF-V进行整合，成功制备了具有分级孔隙的核壳型MOF@COFs复合材料（MIL-68@COF-V）。通过以具有可比厚度的SPME涂层形式，对MIL-68（In）、COF-V以及10%-MIL-68@COF-V对目标分析物的富集性能进行比较。结果表明，MIL-68@COF-V由于整合了MIL-68（In）和COF-V的孔隙结构和较大的比表面积，对氯/溴取代的有机污染物的富集性能优于单一的MIL-68（In）或COF-V。以10%-MIL-68@COF-V作为SPME涂层，建立了一种结合GC-MS的超灵敏分析检测方法，用于海水中的7种氯/溴取代有机污染物的测定（图4）。该工作为利用核壳型MOF@COF材料富集新污染物提供了新见解，并促进了其在环境监测中的应用。针对COFs合成过程中形态/结构不佳而导致COFs易堆叠/聚集的问题，Wu等^［43］^通过在Ti_3_C_2_T *_x_* MXene纳米片上组装*β*-酮胺连接的COFs，制备了一种MXene-COF复合材料（Ti_3_C_2_T *_x_* /TAPT-TFP），该材料兼具MXene的二维结构和出色的吸附能力以及COFs的多级孔隙结构和大比表面积。随后，建立了一种基于Ti_3_C_2_T *_x_* /TAPT-TFP的SPME方法并结合GC-MS技术，对水果、蔬菜中的有机氯农药进行分析检测，该方法具有低检出限（0.036~0.126 ng/g），宽线性范围（0.12~20.0 ng/g）和良好的重复性（4.1%~11.2%）。

**图4:**
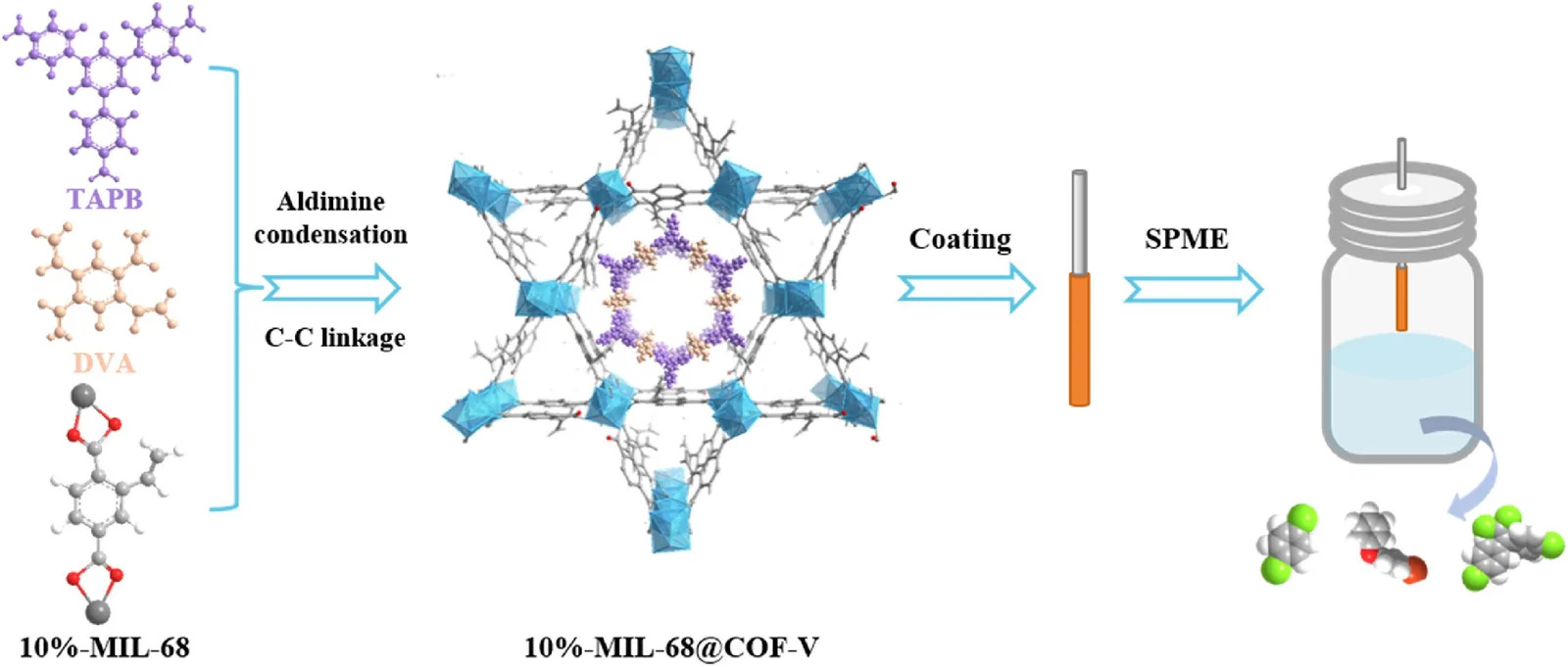
功能化10%-MIL-68@COF-V的合成和SPME流程图^［42］^

### 1.3 HOFs及其复合材料

HOFs是一类由有机或金属-有机构建单元通过氢键连接而成的一类晶态多孔材料。HOFs具有氢键键能弱和方向性低的特点，仅需改变反应条件和溶剂比例就可制备种类丰富的HOFs。目前，HOFs因其合成条件温和、易于重复利用等特点，在新污染物的吸附去除领域展现出良好的应用前景^［44，45］^。Yang等^［46］^通过蒸汽扩散法成功制备了多孔HOF（PFC-1），并将其用于去除水中的两种有机染料罗丹明B（RhB）和甲基橙（MO）。在298.15 K时，PFC-1对RhB和MO表现出出色的去除能力，最大吸附量高达252~317 mg/g。通过DFT计算与多种表征技术，证明了氢键相互作用、静电吸引力和*π*-*π*相互作用是RhB在PFC-1上吸附的主要原因，而范德华力在PFC-1吸附MO中起到了重要作用。PFC-1为HOFs提供了新的应用可能性，证明其在环境修复领域具有巨大的潜力。Jiang等^［47］^报道的氨基功能化HOF（PFC-1-NH_2_）与海藻酸钠（SA）复合形成的PFC-1-NH_2_@SA材料能够实现孔雀石绿的高效和高容量吸附，吸附能力可达到1 086.72 mg/g。通过材料吸附对比实验验证了氨基修饰策略不仅增加了材料吸附位点数量，还增强了与孔雀石绿之间的静电吸引、*π*-*π*堆积和氢键作用。该研究工作表明，HOFs在新污染物的样品前处理中具有良好的应用前景，为富集新污染物提供了新的材料选择（图5a）。Hu等^［48］^通过1，3，6，8-四（4-羧基苯基）芘（H_4_TBAPy）自身及其与三聚氰胺（MA）之间良好的亲和力，在室温下采用温和的溶剂蒸汽扩散法，成功制备了由H_4_TBAPy自组装而成的HOFs复合材料（MA/PFC-1）。MA/PFC-1兼具高比表面积、分级微孔结构、强氢键网络以及丰富的含氮/氧杂原子的功能基团。新引入的MA可进一步提供氢键位点并增强材料的整体极性，这些特性使其成为理想的SPME涂层材料（图5b）。基于此，该团队开发了一种基于MA/PFC-1的SPME涂层与GC-MS联用的分析方法，用于硝基芳香族化合物（NACs）的检测。该方法具有较低的检出限（4.30~20.83 ng/L）和良好的重现性（RSD<8.6%），并成功应用于实际水样中痕量NACs的测定，加标回收率为80.1%~119%。Tian等^［49］^制备了一种磁性功能化HOF材料（Fe_3_O_4_@PFC-1_1/1_）。该材料具有开放的孔隙结构和丰富的结合位点，有利于BPs进入其孔道，并通过范德华力、*π*-*π*相互作用和氢键相互作用等多种作用力与其结合（图5c）。值得注意的是，Fe_3_O_4_@PFC-1_1/1_对多种BPs均表现出良好的吸附性能（249.95~676.06 mg/g）和极快（10 s）的吸附效率。将其作为MSPE吸附剂，与HPLC-DAD联用，开发了一种用于环境与人体样本中痕量BPs的分析方法。该工作表明，将HOFs与磁性纳米颗粒相结合，有望构建一种用于复杂基质中痕量污染物高效富集的吸附剂平台。

**图5:**
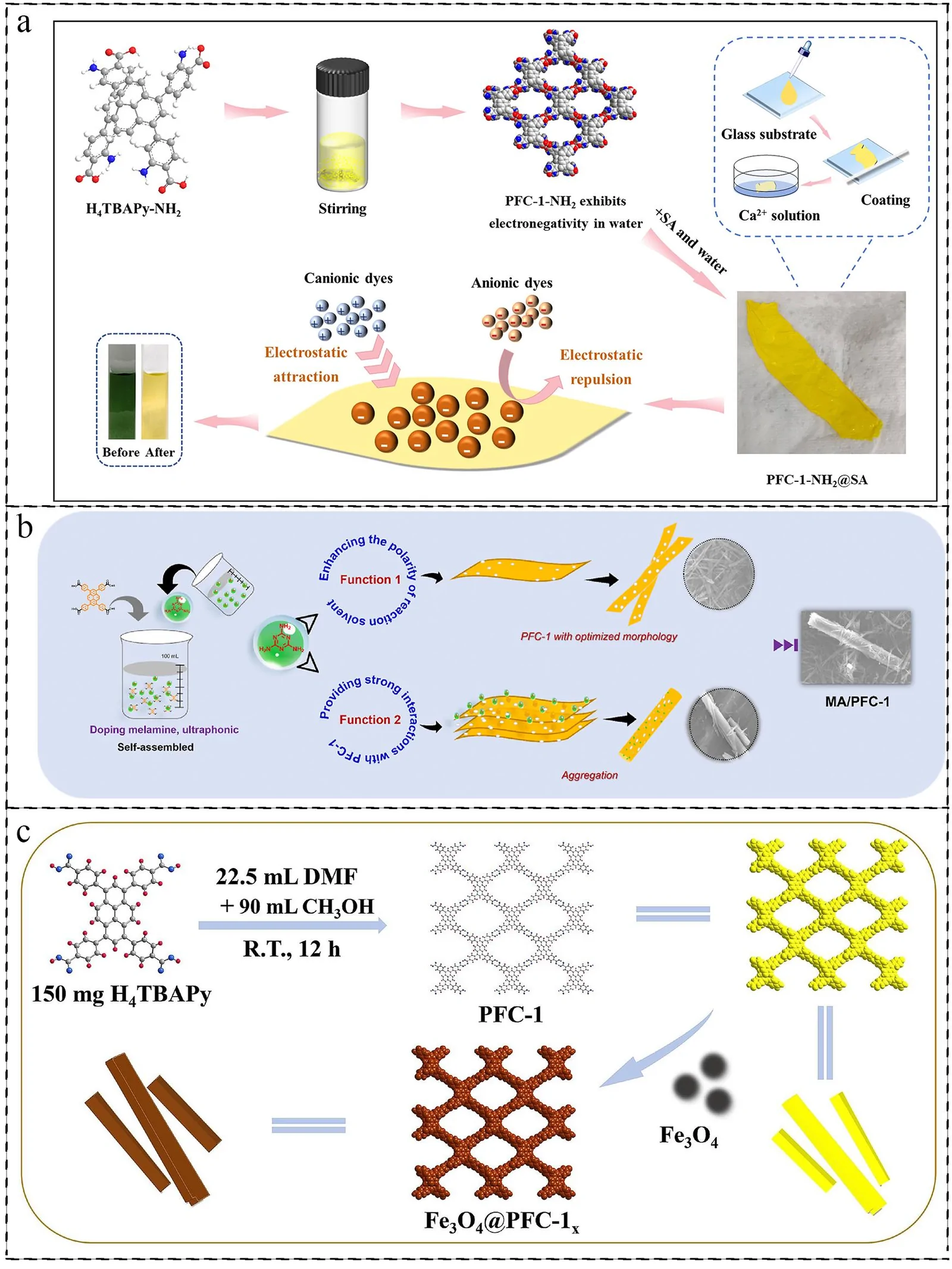
（a）PFC-1-NH_2_@SA用于染料吸附的设计示意图^［47］^；（b）MA/PFC-1的制备流程图^［48］^；（c）Fe_3_O_4_@PFC-1_X_的制备合成图^［49］^

作为一种新兴的多孔框架材料，HOFs在样品前处理中展现出良好的应用潜力，但其进一步发展仍然面临挑战。氢键相互作用的固有强度较弱，导致HOFs的化学稳定性与机械强度不足，这在一定程度上限制了它们在实际样品前处理中的深入应用。此外，如何实现其结构的精准功能化、发展可控的复合材料制备策略，从而在提升吸附性能的同时拓展其应用范围，仍是当前亟需解决的关键科学问题^［50］^。

## 2 多孔框架材料在新污染物分析中的应用

分析食品中新污染物的主要难点在于新污染物浓度较低，常处于痕量甚至超痕量水平，同时食品中基质成分复杂，存在大量干扰性物质。因此，发展高效的样品前处理技术是提升检测灵敏度的关键，其核心在于开发性能优异的新型吸附材料。目前，以MOFs、COFs和HOFs为代表的多孔框架材料，因其具有比表面积大、孔隙率高、化学性质可调等优点，已被广泛应用于新污染物的选择性富集与萃取。

### 2.1 内分泌干扰物

EDCs是一类能干扰生物体内内分泌系统中激素的合成、转运和代谢等环节，从而导致生物体内多种功能异常效应的外源性物质^［51］^。根据其来源，主要可分为天然激素类和人工合成化合物两大类^［52］^。目前，备受关注的EDCs包括双酚类、烷基酚类及激素类等。Wang等^［53］^成功制备了具有多层级结构的磁性复合材料Fe_3_O_4_@NH_2_-MIL-88（Fe）@TpPa，通过NH_2_-MIL-88（Fe）和TpPa之间的协同作用，有效提升了复合材料对BPs的吸附性能。吸附动力学与吸附等温线实验数据表明，吸附剂与BPs之间的吸附作用以多层非均匀化学吸附为主导。建立了基于该复合材料的MSPE-HPLC-UV分析方法，实现了牛奶中4种BPs的高灵敏度检测，回收率为83.7%~100.9%，相对标准偏差低于6.3%，表明该方法具有良好的准确性与重现性（图6a）。Li等^［54］^通过后修饰合成策略，将离子液体（1-五氟苯基-3-乙烯基咪唑溴化物）接枝到MOF（MIL-88（Fe）-NH_2_）上，成功制备了复合材料MIL-88（Fe）-NH_2_-IL。离子液体的引入有效增加了材料的吸附位点，从而提升了其对BPs的吸附能力。以该材料作为吸附剂所建立的dSPE-HPLC-DAD检测方法已经成功应用于蜂蜜和水中多种BPs的测定。该方法的回收率良好（80.3%~113.2%），检出限低（0.05~0.15 μg/L），萃取时间短（3 min），富集因子高（214~354）。该研究工作证明离子液体功能化的MOFs能增强其与目标分析物的多重相互作用能力，为复杂样品中痕量新污染物的精准检测提供了一种技术方案。Jiang等^［55］^报道了一系列卤素（Cl、 Br、 I）功能化的磁性COF（Fe_3_O_4_@COF），系统研究了其对多种BPs的吸附能力。结果表明Fe_3_O_4_@COF@Br对BPs的吸附效果最佳。基于此，将Fe_3_O_4_@COF@Br作为磁性固相萃取吸附剂，结合HPLC-PDA检测技术建立了一种用于食品样品中5种BPs的分析检测方法。DFT计算结果表明，在COFs中引入卤素原子可有效增加其结构中苯环上的电子云密度，从而增强了COFs与BPs之间的*π*-*π*相互作用。同时，卤素原子的引入为COFs与目标分析物之间提供了卤键作用位点。这两种相互作用显著提升了COFs对分析物的吸附含量，该工作从机理上证明了卤素功能化是一种提升材料吸附性能的有效策略。COFs传统的刚性芳香结构因其有限的分子识别能力，制约了对BPs的吸附效果。因此，Su等^［56］^通过2，3-二羟基二羧酸肼（TAH）和1，3，5-三（4-甲酰基苯基）三嗪（TFT）的聚合反应设计制备了一种羟基功能化的柔性脂肪族COFs（TFT-TAH COF），用于高效提取和灵敏检测BPs。这种柔性脂肪族连接单元暴露了更多的活性位点，从而显示出优异的吸附能力（654~1 111 mg/g）和快速吸附动力学（30 s）。通过尺寸匹配、氢键和*π*-*π*堆积的协同作用，TFT-TAH COF对BPs具有良好的亲和力。通过建立了以TFT-TAH COF为吸附剂的dSPE-HPLC联用方法，成功实现了食品样品中BPs的测定，检出限低至0.03~0.1 μg/L。Lian等^［25］^在室温下合成了氮原子含量递增的一系列COFs材料，并将其用于雌激素的SBSE中。其中，N含量最高的TAPT-TA-COF对雌激素表现出最佳的吸附效率，根据DFT计算结果，这种优异吸附能力归因于其合适的孔道结构以及与雌激素之间的氢键和*π*-*π*相互作用。基于该材料建立的TAPT-TA-COF-SBSE-HPLC-DAD方法成功应用于乳制品中雌激素的分析，检出限为0.06~0.15 μg/L（图6b）。该研究工作证明了COFs中氮原子数量与其吸附雌激素效率之间的关系，为针对新污染物的COFs功能化设计提供了科学指导。Xue等^［57］^报道了一种基于MOFs的磁性纳米粒子（Fe_3_O_4_@SiO_2_@MIL-53（Al）-NH_2_），该材料具有制备过程简单且环保绿色、比表面积高、稳定性良好的特点。随后，以其作为吸附剂，建立了一种MSPE-HPLC-DAD检测方法，用于检测鱼肉中的6种雌激素。该方法具有较低的检出限（0.091~0.44 μg/L）、较宽的线性范围（2~5 000 μg/L）和较高的萃取效率（79.3%~115.2%和81.1%~117.8%），展现了在复杂基质中测定雌激素的良好应用前景。本课题组^［58］^通过Schiff-Base缩合反应在MXene表面原位生长COF，构建了一种MXene-COF复合材料。该材料结合了MXene的二维结构和高吸附性能，以及COF的大比表面积与多级孔道结构优势，可实现对类固醇激素的高效吸附。将MXene-COF复合材料作为dSPE吸附剂，结合HPLC-PDA，建立了一种用于畜产品中类固醇激素选择性富集与定量分析的新方法，该方法具有良好的回收率（79.4%~109.9%）、较低的相对标准偏差（0.1%~6.1%），展现了COFs复合材料在应对复杂食品样品前处理挑战中的应用潜力。

**图6:**
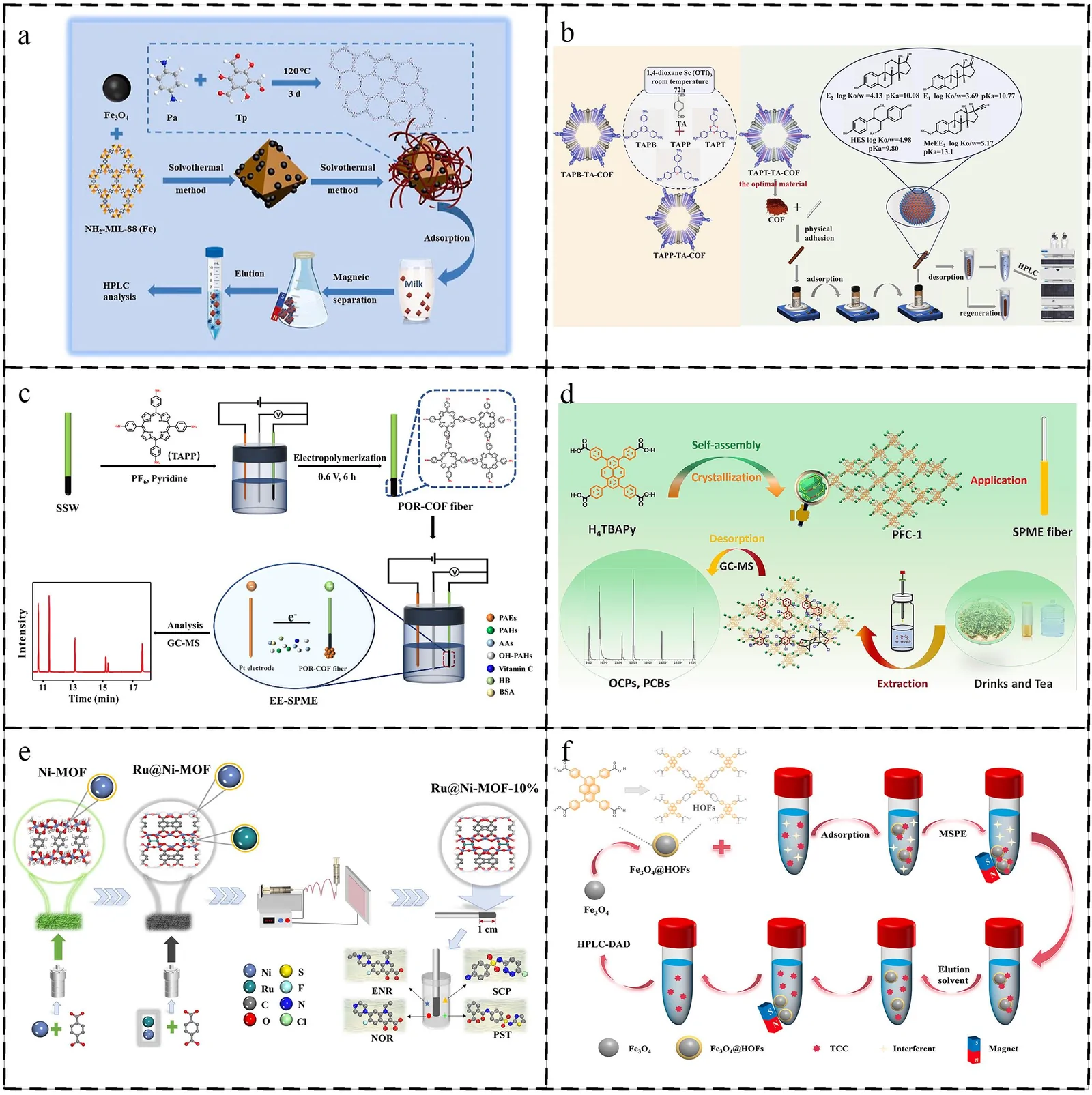
（a）Fe_3_O_4_@NH_2_-MIL-88（Fe）@TpPa的合成及MSPE示意图^［53］^；（b）COF涂层搅拌棒的制备及SBSE操作流程^［25］^；（c）POR-COF 的合成及EE-SPME流程示意图^［59］^；（d）PFC-1纤维的制备及其与GC-MS联用分析OCPs和PCBs的示意图^［60］^；（e）Ru@Ni-MOF用于选择性抗生素提取的合成及应用示意图^［61］^；（f）Fe_3_O_4_@HOFs的制备及四环素的MSPE流程示意图^［62］^

### 2.2 持久性有机污染物

POPs是指具有生物蓄积性、环境持久性和远距离环境迁移的特性，并对生态环境及人类健康产生严重危害的有机化学物质。常见的POPs包括邻苯二甲酸酯类（PAEs）、PFAS、多氯联苯（PCBs）、多环芳烃（PAHs）、有机氯农药（OCPs）等^［63］^。Hosseini等^［64］^采用水热法制备了三元金属有机框架（NiCoMn-MOFs），该材料利用多元金属间的协同效应提升了其吸附性能。将其作为吸附剂，建立了果汁中4种PAEs的dSPE-GC-FLD分析方法，该方法具有高富集因子（325~375）、较低的检出限和定量限（0.11~0.5 ng/L和0.36~0.96 ng/L）、较宽的线性范围（1~250 ng/L）和良好的回收率（82%~106%）。Chen等^［59］^通过一步电聚合的策略，在纤维表面原位构建COF涂层（POR-COF），将其应用于电增强固相微萃取（EE-SPME），与GC-MS/MS联用，建立了牡蛎、饮料等食品样品中6种PAEs的高效、高灵敏的分析检测方法，该策略通过电化学辅助显著提升了萃取效率，为复杂食品基质中痕量污染物的灵敏检测提供了新途径（图6c）。Yeh等^［65］^通过Schiff-Base反应制备了含三氟甲基的*β*-酮烯胺型COF（Tp-BD-CF_3_）。该材料具有出色的化学稳定性，凭借氟-氟、疏水、氢键和静电吸引等多重相互作用，对PFAS展现出快速的吸附动力学（1 min）。将该材料作为吸附剂，并结合超高效液相色谱-MS/MS，建立了一种检测谷物样品中11种PFAS的方法。该研究通过精准的氟功能化设计，实现了对PFAS的快速、高选择性富集，为PFAS的高通量分析提供了可靠方法。Hu等^［60］^通过室温溶剂蒸发法，制备了由1，3，6，8-四（4-羧基苯基）芘自组装而成的HOF材料（PFC-1）。该材料具有优异的热化学稳定性、高比表面积、宽孔径分布及独特的卷曲片状结构，是理想的多功能SPME涂层。基于此，该团队将其用作SPME涂层并与GC-MS联用，建立了适用于水样和茶叶样品中多种POPs（PAHs、OCPs、PAEs等）的高灵敏度分析方法（图6d）。该工作通过HOFs涂层的多重作用机制，实现了对多类POPs的广谱高效富集，拓展了HOFs在食品与环境痕量新污染物分析中的应用维度。

### 2.3 抗生素

抗生素是由微生物产生或人工合成的化学物质，能够抑制或杀灭病原微生物，常见的包括磺胺类、氟喹诺酮类、四环素类等。抗生素被广泛应用于人类与动物的疾病治疗及畜牧业养殖中^［66］^。然而，其过度甚至泛滥使用导致了耐药菌的出现与扩散，严重削弱了临床疗效。食品供应作为连接生产、消费与环境的关键枢纽，已成为抗生素耐药性传播的重要途径之一^［67］^。长期通过膳食摄入含抗生素残留的食品，可能扰乱人体肠道菌群微生态的平衡，从而加剧耐药菌的定植和传播的风险，对公共健康造成严重威胁^［68］^。Tian等^［69］^通过调制剂诱导缺陷形成的策略合成了一系列MOF-525-X材料，实验证明该策略的引入能显著提高MOF对磺胺类抗生素的富集能力，其中MOF-525-6展现出最佳吸附性能（225.07~1 173.22 mg/g），通过多种表征数据和DFT计算结果，证明了吸附机制主要为氢键相互作用和范德华力，而配位作用发挥了协同效应。该材料凸显了该策略在精准调控MOFs吸附性能方面的应用潜力。基于此，该研究建立了以MOF-525-6为吸附剂的dSPE-HPLC-DAD检测方法，成功应用于肉蛋奶中的5种磺胺类抗生素的检测。Li等^［61］^通过一锅法制备了过渡金属掺杂的纳米片状二维双金属Ru@Ni-MOF材料，其中Ru的掺杂优化了Ni活性中心的电子结构，增强了其对氟喹诺酮类和磺胺类抗生素的吸附选择性，表明在MOFs中掺杂过渡金属能有效提升其吸附选择性。通过静电纺丝技术将Ru@Ni-MOF-10%用作SPME涂层，该涂层稳定性高，可重复使用超过200次，结合HPLC-UV实现了牛奶和猪肉中4种抗生素的超痕量检测，检出限为0.002~0.007 μg/L，线性范围为0.005~5 μg/L（图6e）。在COFs上进行分子印迹可提高其对目标分析物的特异性识别和选择性吸附性能，Su等^［70］^采用表面印迹法在COF上构建了以恩诺沙星为模板的分子印迹孔穴，制备了新型吸附剂MI-COF，其对氟喹诺酮类抗生素的吸附容量达到581 mg/g，是非分子印迹COF的2.2倍；证明分子印迹技术可有效赋予COFs特异性识别能力，是提高吸附选择性的理性设计之一。所开发的MI-COF-HPLC方法成功应用于食品样本中氟喹诺酮类抗生素的高灵敏度和高选择性分析。调节COFs的孔径是有利于改善氟喹诺酮类抗生素吸附性能的策略之一，Ao等^［71］^制备了一系列不同孔径的磺酸化COFs，其中，由Tp与4，4′-二氨基-3，3′-联苯二磺酸（BdS）制备而成的磺酸化COF（TBS）材料可以有效减少氟喹诺酮类抗生素的扩散阻力，吸附能力高达627.3 mg/g。基于TBS建立的TBS-SPE-HPLC-FLD方法，成功应用于6种食品样品中氟喹诺酮类抗生素的分析检测。该研究表明，精确调控COFs孔径是提高吸附容量与优化目标分析物的传质效率的有效策略。Li等^［62］^通过原位自组装法在羧基化Fe_3_O_4_表面生长HOF壳层，制备了Fe_3_O_4_@HOFs磁性复合材料，其通过氢键相互作用、*π*-*π*相互作用和疏水相互作用高效吸附四环素。所开发的Fe_3_O_4_@HOFs-MSPE-HPLC方法具有吸附剂用量少（2 mg），萃取效率高（82.4%~103.7%）和线性范围宽（1.0~6.0×10^3^ μg/L）等优点，并成功应用于牛奶、胡萝卜等实际食品样品中四环素的检测，证实了HOFs在样品前处理中的潜力（图6f）。

## 3 总结与展望

多孔框架材料在新污染物的分析检测中发挥着重要的作用，本文围绕MOFs、COFs和HOFs及其复合材料，介绍了如何通过理性设计策略（如配体功能化、缺陷工程、多元复合等）以增强它们吸附性能的研究进展，并详述了其作为高效吸附剂在新污染物样品前处理中的实际应用。首先，介绍了MOFs、COFs和HOFs及其复合材料的类型，阐述了其通过孔径筛分、非共价相互作用及配位作用等多种机制提升对目标新污染物吸附能力的研究现状。在此基础上，针对EDCs、POPs和抗生素等不同类型的新污染物，简要综述了近3年来相关材料在食品基质中新污染物的选择性富集与分析检测方面的应用进展。本文为面向新污染物分析的多孔框架材料吸附剂的后续开发提供了重要的理论依据与技术参考。目前，虽然多孔框架材料在新污染物的样品前处理中已展现出显著优势，但其仍然面临诸多挑战。例如，部分MOFs在极端pH条件下的化学稳定性仍有不足；部分COFs的合成常涉及高温条件或者长时间反应，其规模化制备严重受限；HOFs的结构稳定性普遍依赖于氢键相互作用，在实际复杂样品基质中易发生解离或重组。此外，多数研究仍局限于实验室条件下的标准溶液体系及加标实际样品分析，吸附剂在真实复杂食品基质中的实际应用性能仍需进一步系统评估与验证。未来的研究可重点关注以下几个方面：第一，深度融合理论计算与原位表征技术，在分子水平上阐明吸附剂结构及其性能的构效关系，尤其应阐明复杂食品基质，如脂质、蛋白质等共存时吸附剂与目标分析物的相互作用机制，以指导具有特异性识别功能的吸附材料的理性设计。第二，发展绿色、可持续的合成路径。如以生物质为原料或开发新型绿色溶剂，并着力提升温和条件下制备得到的HOFs的稳定性，以拓展其应用范围。第三，通过复合材料设计与表面功能化等策略，提升材料在实际复杂基质中的化学稳定性、机械强度和重复使用性。第四，推动材料向实用化器件集成。例如开发稳定的SPME涂层或自支撑复合薄膜，并探索其与现有分析仪器的在线、自动化联用技术。第五，发展以问题为导向的材料理性设计。例如，针对MOFs材料在酸性或水性食品基质（如果汁、醋等）中易水解的问题，或可采用疏水涂层、聚合物封装等方式保护材料的结构稳定性。针对COFs材料在动物组织等高脂食品中易被非特异性吸附污染的问题，可通过精确调控孔径与表面极性，实现物理筛分与化学识别的协同保护作用。

## References

[1] Wang Y W， Zhang Q R， Yu N Y， et al . Progress in Chemistry， 2024， 36（11）： 1607

[2] Zhao X， Wang X， He J， et al . Nat Commun， 2025， 16（1）： 7775 10.1038/s41467-025-63217-4 PMC12367988 40835613

[3] Kaur I， Batra V， Kumar Reddy Bogireddy N， et al . Food Chem， 2023， 406： 135029 10.1016/j.foodchem.2022.135029 36463597

[4] Peivasteh-Roudsari L， Barzegar-Bafrouei R， Sharifi K A， et al . Heliyon， 2023， 9（7）： e18140 10.1016/j.heliyon.2023.e18140 PMC10395372 37539203

[5] Zheng J， Tian L， Bayen S . Crit Rev Food Sci Nutr， 2023， 63（16）： 2687 10.1080/10408398.2021.1980369 34583591

[6] González N， Marquès M， Nadal M， et al . Food Chem Toxicol， 2019， 125： 370 10.1016/j.fct.2019.01.021 30682385

[7] Sun Y， Su B， Zhang K， et al . Food Chem， 2025， 489： 144986 10.1016/j.foodchem.2025.144986 40466536

[8] Kang J Y， Zhao X B， Shi Y P . ACS Appl Mater Interfaces， 2023， 15（36）： 42981 10.1021/acsami.3c08703 37642085

[9] Liu C， Wang X， Wang Q， et al . Food Chem， 2026， 504： 147968 10.1016/j.foodchem.2026.147968 41548269

[10] Hu Z， Yang Y， Li Z， et al . Talanta， 2025， 287： 127667 10.1016/j.talanta.2025.127667 39892117

[11] Meng L S， Wang X L， Wang X， et al . Food Chem， 2024， 447： 138867 10.1016/j.foodchem.2024.138867 38447237

[12] Liu C， Wang X， Wang Q， et al . Chem Eng J， 2026， 527： 171313

[13] Sokołowski A， Dybowski M P， Oleszczuk P， et al . Food Chem， 2024， 440： 138222 10.1016/j.foodchem.2023.138222 38134829

[14] Li J， Wang Z， Li J， et al . Food Chem， 2022， 388： 133007 10.1016/j.foodchem.2022.133007 35483283

[15] Liang Y， Qi Y， Zhong N， et al . Coord Chem Rev， 2025， 533： 216507

[16] Chen J， Bae B， Lee J . TrAC-Trends Anal Chem， 2026， 195： 118547

[17] Ding D， Wang Y， He J， et al . Environ Toxicol Chem， 2025， 44（5）： 1165 10.1093/etojnl/vgaf032 39878890

[18] Marchel M， Rayaroth M P， Wang C， et al . Chem Eng J， 2023， 475： 144971

[19] Liu H Y， Jin J， Guo C C， et al . Chinese Journal of Chromatography， 2021， 39（8）： 835 10.3724/SP.J.1123.2021.02035 PMC9404209 34212584

[20] Jiang L S， Zhou Q X . Chinese Journal of Chromatography， 2025， 43（2）： 107 10.3724/SP.J.1123.2024.07020 PMC11758229 39844701

[21] Liu Z， Wang J， Duan T， et al . J Chromatogr A， 2022， 1679： 463387 10.1016/j.chroma.2022.463387 35933771

[22] Yang X， Wang J， Chang G， et al . Food Chem， 2024， 439： 138182 10.1016/j.foodchem.2023.138182 38100880

[23] Wang P， Ma J P， Li S， et al . Chinese Journal of Chromatography， 2024， 42（3）： 264 10.3724/SP.J.1123.2023.05012 PMC10955369 38503703

[24] Kang J Y， Zhao X B， Huang J Q， et al . Chem Eng J， 2025， 518： 164858

[25] Lian X， Wang L， Sun J， et al . Food Chem， 2025， 492： 145616 10.1016/j.foodchem.2025.145616 40716401

[26] Li Y， Wei J， Wang J， et al . Chem Soc Rev， 2025， 54（6）： 2693 10.1039/d4cs00591k 39841538

[27] Afridi M N， Adil S， Byambaa B， et al . J Water Process Eng， 2024， 63： 105530

[28] Musarurwa H， Chimuka L， Tavengwa N T . Trends Environ Anal Chem， 2021， 32： e00141

[29] Yaghi O M， Li G， Li H . Nature， 1995， 378（6558）： 703

[30] Cai G， Jiang H-L . Angew Chem Int Ed， 2017， 56（2）： 563 10.1002/anie.201610914 27930852

[31] Han L， Qin P， Li M， et al . Chem Eng J， 2023， 456： 140969

[32] Grancha T， Garcia-Atienza P， Garcia L， et al . Small， 2025， 21（51）： e10000 10.1002/smll.202510000 PMC12723331 41195623

[33] Mao L， Qian J . Small， 2024， 20（20）： 2308732 10.1002/smll.202308732 38072778

[34] Qin P， Zhu S， Mu M， et al . Chin Chem Lett， 2023， 34（12）： 108620

[35] Ilic N， Tan K， Mayr F， et al . Adv Mater， 2025， 37： 2413120 10.1002/adma.202413120 PMC11817902 39573850

[36] Zhou X， Lai X， Hu K . J Chromatogr A， 2025， 1743： 465681 10.1016/j.chroma.2025.465681 39824070

[37] Guo H， Li Y， Li Y， et al . ACS Appl Mater Interfaces， 2023， 15（11）： 14777 10.1021/acsami.2c22386 36897016

[38] Deng Y， Wang Y， Xiao X， et al . Small， 2022， 18（38）： 2202928 10.1002/smll.202202928 35986438

[39] Yang Y， Liu Q， Zou Y， et al . J Environ Chem Eng， 2023， 11（5）： 110975

[40] Li S， Liu W， Wang Q， et al . Food Chem， 2022， 382： 132362 10.1016/j.foodchem.2022.132362 35152018

[41] Tao Q， Tan X， Huang Y， et al . J Colloid Interface Sci， 2026， 702： 138921 10.1016/j.jcis.2025.138921 40939413

[42] Gan L， Feng X， Zhou S， et al . Talanta， 2025， 293： 128091 10.1016/j.talanta.2025.128091 40199122

[43] Wu J， Wang Z， Zhang S， et al . Small， 2024， 20（10）： e2305730 10.1002/smll.202305730 37902412

[44] Zhang Y， Tian M， Majeed Z， et al . Separations， 2023， 10（3）： 196

[45] Yang L， Yuan J， Wang G， et al . Adv Funct Mater， 2023， 33（28）： 2300954

[46] Yang J， Wang J， Zhang X， et al . Micropor Mesopor Mat， 2022， 330： 111624

[47] Jiang Y， He X， Zhu R， et al . J Environ Sci， 2026， 164： 204 10.1016/j.jes.2025.07.025 42070811

[48] Hu Y， Liu Y， Kuang Y， et al . Anal Chim Acta， 2023， 1277： 341652 10.1016/j.aca.2023.341652 37604609

[49] Tian X， Hao Y， Yang J， et al . Chem Eng J， 2024， 500： 157223

[50] Song X， Wang Y， Wang C， et al . J Am Chem Soc， 2022， 144（24）： 10663 10.1021/jacs.2c02598 35675383

[51] Azizi D， Arif A， Blair D， et al . Environ Res， 2022， 207： 112196 10.1016/j.envres.2021.112196 34634314

[52] Nowak K， Jabłońska E， Ratajczak-Wrona W . Environ Int， 2019， 125： 350 10.1016/j.envint.2019.01.078 30743143

[53] Wang Q， Chen L， Cui X， et al . Talanta， 2023， 256： 124268 10.1016/j.talanta.2023.124268 36657241

[54] Li D， Hao Y， Zhou H， et al . Chem Eng J， 2025， 507： 160784

[55] Jiang H， Tao H， Yang S， et al . Food Chem， 2025， 489： 145021 10.1016/j.foodchem.2025.145021 40472515

[56] Su L， Wang L， Zhao L， et al . Talanta， 2026， 299： 129134 10.1016/j.talanta.2025.129134 41252909

[57] Xue B， Xu M， Bai Z， et al . Adv Sample Prep， 2024， 11： 100120

[58] Huang J Q， Han Y， Ha W， et al . J Chromatogr A， 2025， 1766： 466629 10.1016/j.chroma.2025.466629 41418652

[59] Chen H， Wang J， Zhang W， et al . ACS Appl Mater Interfaces， 2023， 15（9）： 12453 10.1021/acsami.3c00580 36826831

[60] Hu Y， Li Y， Shi Y， et al . Food Chem， 2023， 415： 135790 10.1016/j.foodchem.2023.135790 36868067

[61] Li H， Zhang Q， Liu X， et al . Chem Eng J， 2025， 523： 168546

[62] Li Z Y， Zhu J H， Zhao J， et al . Talanta， 2024， 280： 126746 10.1016/j.talanta.2024.126746 39182415

[63] Liu L Y， Ma W L， Jia H L， et al . Environ Pollut， 2016， 217： 70 10.1016/j.envpol.2015.12.056 26775724

[64] Hosseini M， Ghanbarzadeh B， Pezeshki A， et al . J Food Compos Anal， 2025， 140： 107227

[65] Yeh C L， He J Y， Antonia T Z R， et al . Microchem J， 2025， 219： 115955

[66] Okoye C O， Nyaruaba R， Ita R E， et al . Environ Toxicol Pharmacol， 2022， 96： 103995 10.1016/j.etap.2022.103995 36210048

[67] Li J， Song J， Wang X， et al . J Agric Food Chem， 2025， 73（46）： 29310 10.1021/acs.jafc.5c10696 41204637

[68] Chen J， Ying G G， Deng W J . J Agric Food Chem， 2019， 67（27）： 7569 10.1021/acs.jafc.9b01334 31198037

[69] Tian X， Li Y， Ren M， et al .J Hazard Mater， 2026， 501： 140733 10.1016/j.jhazmat.2025.140733 41371141

[70] Su L H， Qian H L， Yang C， et al . J Hazard Mater， 2023， 459： 132031 10.1016/j.jhazmat.2023.132031 37467605

[71] Ao S Q， Liu S P， Jiang Y， et al . Talanta， 2025， 294： 128142 10.1016/j.talanta.2025.128142 40267643

